# Statement of Retraction

**DOI:** 10.1080/10717544.2021.1868133

**Published:** 2021-01-20

We, the Editors and Publishers of *Drug Delivery*, have retracted the following article:

Niyaz Ahmad, Iqbal Ahmad, Sadiq Umar, Zeenat Iqbal, Mohd Samim & Farhan Jalees Ahmad (2016) PNIPAM nanoparticles for targeted and enhanced nose-to-brain delivery of curcuminoids: UPLC/ESI-Q-ToF-MS/MS-based pharmacokinetics and pharmacodynamic evaluation in cerebral ischemia model, *Drug Delivery*, 23:7, DOI: 10.3109/10717544.2014.941076

This article is retracted due to reuse of images from other articles by the same authors, representing results of different experiments. This includes Figure 1(B), which can also be found in the following article, where it’s presented, after contrast and frame adjustments, as Figure 10(c):

- Ahmad et al. (2017) Isolation, characterization, and quantification of curcuminoids and their comparative effects in cerebral ischemia*, Journal of Liquid Chromatography & Related Technologies*, DOI: 10.1080/10826076.2017.1293549

In addition, various panels from Figure 10 were reused in other articles, after color adjustments, as follows:

- Figure 10(a) is presented as Figure 10(d) in: Ahmad et al. (2019), Brain-targeted glycyrrhizic-acid-loaded surface decorated nanoparticles for treatment of cerebral ischemia and its toxicity assessment, *Artificial Cells and Nanotechnology,* DOI: 10.1080/21691401.2018.1561458
- Figure 10(a) is presented as Figure 11(b) in: Ahmad et al (2020), Poloxamer-chitosan-based Naringenin nanoformulation used in brain targeting for the treatment of cerebral ischemia, *Saudi Journal of Biological Sciences*, DOI: 10.1016/j.sjbs.2019.11.008
- Figure 10(b) is presented as Figure 10(f) in: Ahmad et al. (2019), Brain-targeted glycyrrhizic-acid-loaded surface decorated nanoparticles for treatment of cerebral ischemia and its toxicity assessment, *Artificial Cells and Nanotechnology,* DOI: 10.1080/21691401.2018.1561458
- Figure 10(e) is presented as Figure 10(b), and an excerpt of Figure 10(g) is presented as one of the mirror-image sides of Figure 10(c) in: Ahmad et al. (2019), Brain-targeted glycyrrhizic-acid-loaded surface decorated nanoparticles for treatment of cerebral ischemia and its toxicity assessment, *Artificial Cells and Nanotechnology*, DOI: 10.1080/21691401.2018.1561458

The authors have not responded to our requests to provide an explanation or to provide the original images. Because of this, the editor agreed the findings of the study are no longer valid and advised for the article to be retracted.

We have been informed in our decision-making by our policy on publishing ethics and integrity and the COPE guidelines on retractions.

The retracted article will remain online to maintain the scholarly record, but it will be digitally watermarked on each page as “Retracted”.

